# Clinicopathologic characteristics of early-onset breast cancer: a comparative analysis of cases from across Ghana

**DOI:** 10.1186/s12905-022-02142-w

**Published:** 2023-01-03

**Authors:** Patrick Kafui Akakpo, Emmanuel Gustav Imbeah, Lawrence Edusei, Simon Naporo, Kofi Ulzen-Appiah, Joe Nat Clegg-Lamptey, Florence Dedey, Josephine Nsaful, Nelson Affram, Beatrice Wiafe, Samuel Mensah, Michael Nortey, Mohammed Sheriff, Forster Amponsah-Manu, Kwabena Agbedinu, Evelyn Mawunyo Jiagge

**Affiliations:** 1 Department of Pathology, University of Cape Coast/Cape Coast Teaching Hospital, Cape Coast, Ghana; 2 ACT Pathology Consult, Pedu Estate, Cape Coast, Ghana; 3 Pathologists Without Borders Ltd, A585/4 Eduardo Mohdlana St. Laterbiokoshie, Accra, Ghana; 4 Department of Surgery, University of Ghana Medical School/Korle Bu Teaching Hospital Accra, Accra, Ghana; 5 Department of Surgery, Ho Teaching Hospital, Ho, Ghana; 6 Peace and Love Hospital, Kumasi, Ghana; 7 Department of Surgery, University of Cape Coast / Cape Coast Teaching Hospital, Cape Coast, Ghana; 8 Department of Surgery, Tamale Teaching Hospital, Tamale, Ghana; 9 Department of Surgery, Eastern Regional Hospital, Koforidua, Ghana; 10 Department of Surgery, Komfo-Anokye Teaching Hospital, Kumasi, Ghana; 11 Henry Ford Cancer Institute/Henry Ford Health System, 2799W Grand Blvd, Detroit, MI USA; 12 Department of Surgery, School of Medicine and Dentistry, Kwame Nkrumah University of Science and Technology, Kumasi, Ghana

**Keywords:** Breast cancer, Early-onset, Pathology, Characteristics, Prognosis, Ghana

## Abstract

**Background:**

Breast cancer is the commonest cancer diagnosed globally and the second leading cause of cancer-related mortality among women younger than 40 years. This study comparatively reviewed the demographic, pathologic and molecular features of Early-Onset Breast Cancer (EOBC) reported in Ghana in relation to Late Onset Breast Cancer (LOBC).

**Methods:**

A descriptive, cross-sectional design was used, with purposive sampling of retrospective histopathology data from 2019 to 2021. Reports of core or incision biopsy, Wide Local Excision or Mastectomy with or without axillary lymph node dissection specimen and matched immunohistochemistry reports were merged into a single file and analysed with SPSS v. 20.0. Descriptive statistics of frequencies and percentages were used to describe categorical variables. Cross-tabulation and chi-square test was done at a 95% confidence interval with significance established at *p* < 0.05.

**Results:**

A total of 2418 cases were included in the study with 20.2% (488 cases) being EOBCs and 79.8% (1930 cases) being LOBCs. The median age at diagnosis was 34.66 (IQR: 5.55) in the EOBC group (< 40 years) and 54.29 (IQR: 16.86) in the LOBC group (≥ 40 years). Invasive carcinoma—No Special Type was the commonest tumour type with grade III tumours being the commonest in both categories of patients. Perineural invasion was the only statistically significant pathologic parameter with age. EOBC was associated with higher DCIS component (24.8% vs 21.6%), lower hormone-receptor-positive status (52.30% vs 55.70%), higher proliferation index (Ki-67 > 20: 82.40% vs 80.30%) and a higher number of involved lymph nodes (13.80% vs 9.00%). Triple-Negative Breast cancer (26.40% vs 24.30%) was the most predominant molecular subtype of EOBC.

**Conclusion:**

EOBCs in our setting are generally more aggressive with poorer prognostic histopathological and molecular features when compared with LOBCs. A larger study is recommended to identify the association between relevant pathological features and early onset breast cancer in Ghana. Again, further molecular and genetic studies to understand the molecular genetic drivers of the general poorer pathological features of EOBCs and its relation to patient outcome in our setting is needed.

## Introduction

Globally, breast cancer is currently the leading cancer diagnosed across all ages and sexes with an incidence of 11.7% (approximately 2.3 million cases) [1]. It is the fifth cause of cancer-related mortality and accounts for 6.9% (684,996 deaths) of deaths among both sexes and all age groups [1]. According to GLOBOCAN 2020 statistics, breast cancer accounted for 17% (247,953 cases) of all diagnosed malignancies among persons younger than 40 years, making it the leading cancer and the second leading cause of cancer-related death among this age group. It accounts for 9.2% (42 767) of cancer deaths in Africa [1]. In Ghana, 19.5% (1147 cases) of all newly diagnosed cancer in 2020 were breast cancers. With a prevalence of 15.2% (1938 cases), it is the commonest cancer diagnosed and accounts for 13.6% (440 cases) of all cancer-related death, second only to liver cancer which accounted for 23.2% (752) of cancer deaths among this age group [1].

Although breast cancer is a predominant disease of ageing, Early-Onset Breast Cancer (EOBC) has recently attracted great interest [2]. EOBC generally refers to breast cancer diagnosed in individuals younger than 40 years of age, even though some researchers use different definitions [3]. Among premenopausal women, it has been reported that those under 40 years of age have a higher risk of dying from breast cancer [4].

Studies have linked young age to more aggressive tumour biology, poorer prognosis and worse clinical outcome. EOBC has been reported to be more commonly HER-2 enriched (22%) [3] and have lower frequency of luminal A tumours (17.2% vs 30%) in LOBC. High tumour grade, multifocal disease, and nodal involvement are some of the other histopathological characteristics that are more frequently associated with EOBC [3, 4]. Younger patients are diagnosed at a more advanced stage than older patients. These aggressive characteristics of EOBC correlate with a poorer prognosis and a higher mortality (up to 1.5 times higher) compared to LOBC [5]. In addition, the European Organization for Research and Treatment of Cancer (EORTC) and the National Surgical Adjuvant Breast and Bowel Project (NSABP) concluded that there was a higher risk of local recurrence in patients younger than 35 years [5].

We however do not currently have data on EOBC in Ghanaian women, this study therefore aims to comparatively review the demographic, pathologic and molecular features of Early-Onset Breast Cancer reported in Ghana.

## Methodology

### Study location

The study was conducted with records from the largest pathology service in Ghana. ACT Pathology Consult (spoke) and Pathologists Without Borders (hub). ACT Pathology Consult receives an average of 1500 surgical pathology specimen yearly while Pathologists Without Borders (PWB) receives an average of 8500 surgical pathology specimen yearly. PWB runs in-house immunohistochemistry (IHC) all year round and serves as the hub for ACT Pathology Consult. Combined, their main catchment area includes the Western, Central, Greater Accra, Eastern, Ashanti, Northern and Volta regions of Ghana. The laboratory, however receives specimens from as far as the Upper West region of Ghana. PWB reports the largest proportion of breast cancer cases in Ghana. The study was conducted with Precision Medicine for Aggressive Breast Cancers (PMABC), which is a research partnership between Henry Ford Health, Detroit, USA, PWB, ACT as well as all the leading Teaching and Regional hospitals in Ghana.

### Study design

This study is descriptive, cross-sectional, and purposive, using retrospective histopathology data retrieved from the electronic medical records system of the reporting centres from January 2019 through to December 2021.

### Data source and sampling

Retrospective data of all breast cancer cases reported at both Pathologists Without Borders and ACT Pathology Consult were derived from the electronic medical records (NubiaEMR v 2.0). The data was exported in an excel file and de-identified. Patient sample type included core and incision biopsies, wide local excision and mastectomy, with or without axillary lymph node dissection. Demographic data was also obtained in addition to matched immunohistochemistry reports. SPSS v 20.0 was used for analysis.

### Inclusion and exclusion criteria

We included data on all patients with histopathologically confirmed breast cancer, and with histopathology and immunohistochemistry reports both present in the electronic database from January 2019 to December 2021. Patients aged below 40 years were grouped as EOBC and those 40 years and above as LOBC. Patients with diagnoses other than breast lesions, benign breast lesions and those with incomplete histopathological reports, no IHC report or incomplete excision biopsy reports were excluded from this study.

### Statistical analysis

In SPSS v 20.0, descriptive statistics of frequencies and percentages were used to describe categorical variables. Cross-tabulation was done to compare variables and chi-square was done to identify statistical relations between variables at a 95% confidence interval with significance established at *p* < 0.05.

## Results

A total of 2418 patients were identified for the study out of which 20.2% (488) were younger than 40 years (EOBC) and 79.8% (1930) were 40 years or older (LOBC).

Demographic data (age and sex) and histopathological parameters (Biopsy type, histological type of tumour, grade, presence or absence of lymphovascular invasion (LVI) and perineural invasion (PNI), presence or absence of carcinoma in-situ (CIS) and type of CIS, hormone receptor (ER, PR) status, HER-2 expression, Ki-67 score, size of tumour and nodal involvement) in both males and females are presented in Table 1. The median age at diagnosis was 34.66 (IQR: 5.55) (488 patients) in the EOBC group (< 40 years) and 54.29 (IQR: 16.86) (1930 patients) in the LOBC group (≥ 40 years). Invasive carcinoma No Special Type (NST) was the commonest tumour type with grade III tumours being the commonest tumour grade in both age groups. LVI and PNI were mostly not identified in both groups. Of the EOBC cases with DCIS present (24.8%, 121 cases), the majority (48.8%, 59 cases) were of intermediate grade. There was a statistically significant relationship between age of patient and the presence of Perineural Invasion (PNI), with LOBCs more likely to show PNI. Tumours in younger patients (EOBC), were characterised by higher presence of DCIS (24.8% vs 21.6%), lower hormone-receptor positive status (52.30% vs 55.70%), higher proliferation index (Ki-67 > 20: 82.40% vs 80.30%) and higher likelihood of nodal involvement (13.80% vs 9.00%) though none of these adverse histopathological features was statistically significant.

**Table 1:** Clinicopathological characteristics of EOBC (< 40 years) and LOBC (≥ 40 years)

	Age group 40 years		*p*-value
	< 40	≥ 40	
*Median (IQR)*	34.66 (5.55)	54.29 (16.86)	
*Sex*			
Male	9 (1.80%)	24 (1.20%)	0.307
Female	479 (98.20%)	1906 (98.80%)	
Total	488 (100%)	1930 (100%)	
*Biopsy type*			
Incision biopsy	29 (5.90%)	128 (6.60%)	0.437
Needle core biopsy	372 (76.20%)	1487 (77.00%)	
Re-excision	2 (0.40%)	5 (0.30%)	
Simple mastectomy/WLE	34 (7.00%)	120 (6.20%)	
WLE/mastectomy + ALND	38 (7.80%)	143 (7.40%)	
Other	13 (2.70%)	47 (2.40%)	
Total	488 (100%)	1930 (100%)	
*Histological type of tumour major*			
DCIS only	22 (4.50%)	51 (2.60%)	0.096
Invasive carcinoma NST	416 (85.20%)	1708 (88.50%)	
Lobular carcinoma	18 (3.70%)	42 (2.20%)	
Mixed invasive carcinoma	8 (1.60%)	30 (1.60%)	
Mucinous carcinoma	15 (3.10%)	52 (2.70%)	
Other	9 (1.80%)	47 (2.40%)	
Total	488 (100%)	1930 (100%)	
*Grade*			
I	90 (18.40%)	297 (15.40%)	0.539
II	154 (31.60%)	675 (35.00%)	
III	231 (47.30%)	912 (47.30%)	
Not assessable	13 (2.70%)	46 (2.40%)	
Total	488 (100%)	1930 (100%)	
LVI*			
Present	75 (15.40%)	295 (15.30%)	0.897
Not identified	403 (82.60%)	1602 (83.00%)	
Possible	10 (2.00%)	33 (1.70%)	
Total	488 (100%)	1930 (100%)	
PNI*			
Present	8 (1.60%)	69 (3.60%)	0.03
Not identified	480 (98.40%)	1861 (96.40%)	
Total	488 (100%)	1930 (100%)	
*DCIS*			
Present	121 (24.80%)	416 (21.60%)	0.124
Not present	367 (75.20%)	1514 (78.40%)	
Total	488 (100%)	1930 (100%)	
*DCIS grade*			
Low grade	26 (21.50%)	75 (18.00%)	0.197
Intermediate	59 (48.80%)	192 (46.20%)	
High grade	36 (29.80%)	149 (35.80%)	
Total	121 (100%)	416 (100%)	
*Hormone receptor status*			
Positive	255 (52.30%)	1075 (55.70%)	0.172
Negative	233 (47.70%)	855 (44.30%)	
Total	488 (100%)	1930 (100%)	
*Ki-67 group (20)*			
< 20	86 (17.60%)	381 (19.70%)	0.29
> 20	402 (82.40%)	1549 (80.30%)	
Total	488 (100%)	1930 (100%)	
*Tumour size group*			
< 2 cm	10 (30.30%)	30 (23.30%)	0.913
2.0–5.0 cm	14 (42.40%)	71 (55.00%)	
> 5.0 cm	9 (27.30%)	28 (21.70%)	
Total	33 (100%)	129 (100%)	
*pT*			
pT0	4 (10.50%)	14 (9.90%)	0.271
pTis (DCIS)	4 (10.50%)	6 (4.30%)	
pTis (Paget)	0 (0.00%)	1 (0.70%)	
pT1	10 (26.30%)	38 (27.00%)	
pT2	14 (36.80%)	49 (34.80%)	
pT3	4 (10.50%)	17 (12.10%)	
pT4	2 (5.30%)	16 (11.30%)	
Total	38 (100%)	141 (100%)	
*pN*			
pN0 (i+)	0 (0.00%)	1 (0.80%)	0.888
pN0	10 (34.50%)	44 (36.10%)	
pN1	10 (34.50%)	33 (27.00%)	
pN2	5 (17.20%)	33 (27.00%)	
pN3	4 (13.80%)	11 (9.00%)	
Total	29 (100%)	122 (100%)	

LVI, lymphovascular invasion; PNI, perineural invasion

Table 2 shows the molecular characteristics of EOBCs and LOBCs. The most predominant molecular subtype of breast cancer among the EOBC group was Triple-Negative Breast Cancer (TNBC) with (26.40% vs 24.30%). The parameter labelled ‘Others’ included cases with HER-2 equivocal results. Among The ‘Other’ category, receptor positivity was seen more frequently in LOBCs than in EOBCs (61.50% against 55.00%). The differences in molecular characteristics however did not reach statistical significance.

**Table 2:** Molecular subtypes of breast cancer in patients younger than 40 years) and forty years or older

	Age group in years		*p*-value
	< 40	≥ 40	
*Molecular subtype*			
Luminal A	55 (11.30%)	209 (10.80%)	0.78
Luminal B (HER2 positive)	46 (9.40%)	182 (9.40%)	0.998
Luminal B (HER2 negative)	93 (19.10%)	427 (22.10%)	0.141
HER2-enriched	54 (11.10%)	225 (11.70%)	0.715
TNBC	129 (26.40%)	469 (24.30%)	0.329
Others	111 (22.70%)	418 (21.70%)	0.604
Total	488 (100%)	1930 (100%)	0.476
*Receptor status versus HER2 equivocal*			
Positive (ER+/PR+, ER−/PR+, ER+/PR−)	61 (55.00%)	257 (61.50%)	0.213
Negative (ER−/PR−)	50 (45.00%)	161 (38.50%)	
Total	111 (100%)	418 (100%)	

ER, estrogen receptor; PR, progesterone receptor; HER-2, human epidermal growth factor receptor-2

## Discussion

This is a large study looking at 2418 Ghanaian breast cancer patients from across the country. The International Agency for Research on Cancer (IARC) estimated the number of prevalent cases (5-years) in 2020 to be 10,134, (21.5% of all cancer cases) with an estimated incidence of 4482 (18.7% of all cancers) in the same year [1]. The estimates also suggest that 1147 (19.7% of breast cancers) were diagnosed in patients younger than 40 years (EOBC) with an estimated number of prevalent cases (5-years) of EOBC in the same year being 15.2% [1]. 20.2% of our study population was EOBC which is higher than the IARC’s estimated number of prevalent cases for the same age group over 5-years (15.2%). The figure is however closer to the prevalence of EOBC for one year (18.6%) and for three (3) years (17.1%). Other researchers have reported a rate of 30% of Ghanaian breast cancer patients [6]. Male breast cancer was more frequent in the EOBC compared to LOBC (1.8% vs 1.2%). The median age of patients with EOBC was 34.66 years. This is lower than the 36 years reported for a study of EOBCs in the UK involving 2956 patients [7]. In relation to age, it is documented that breast cancer in women older than 40 years generally occurs at a lower age in Ghanaians [1, 6]. Our findings thus suggest that this is also true for patients younger than 40 years.

Similar to previous reports, Invasive Carcinoma, No Special Type (NST) / Not Otherwise Specified (NOS) was the commonest type of breast cancer in both EOBC and LOBC patients [6–9]. However, in our cohort, the proportion of Invasive Carcinoma NST/NOS was lower among patients younger than 40 years (85.20%) compared to those 40 years or older (88.50%). Presence of DCIS component was however higher in EOBCs (24.80% vs 21.60%). The occurrence of ‘special types’ of carcinomas such as lobular carcinoma and mucinous carcinoma was just a fraction higher in EOBC compared to LOBC (3.7% and 3.10%) as against (2.20% and 2.70%) respectively. These findings differ from that of studies by Andrikopoulou et al. who found a higher rate of invasive ductal carcinoma in younger patients than in older patients [4]. They however reported similar findings in relation to the overall frequency of invasive ductal carcinomas compared to other cancers such as lobular carcinoma, thus confirming the general established trend that invasive ductal carcinoma is the commonest type of breast cancer diagnosed among all age groups.

Breast cancer has been reported to be generally more aggressive, and of higher grade in Africans [4, 6, 10]. In line with the established trend, majority of diagnosed cancers were of higher grade. The proportions of grade III tumours (47.30%) was the same in both EOBC and LOBC. Though a slightly higher proportion of Grade I tumours (18.40%) were reported in the EOBC group compared to the LOBC group (15.40%), the Ki 67 score of tumours was higher in the EOBC. This conforms to literature that EOBCs express higher levels of the cellular proliferation associated antigen Ki-67 in line with their aggressiveness [11–13].

Though we did not find any significant difference in lymphovascular invasion status between both age categories, there was a more than two-fold chance that there was perineural invasion by cancers in older patients (LOBCs) when compared with EOBCs (3.6% vs 1.60%). This was statistically significant and may be due to the larger number of late onset cancers in our cohort. Again, the relationship between ageing, breast density and susceptibility to perineural invasion in the event of cancer needs more attention [14].

In relation to stage, although majority of cases in both age groups were late stage, many more (27.30%) EOBCs were larger, with size > 5.0 cm compared to 21.7% in the LOBC. The general low rate of screening services in Ghana has been cited as a reason for the late presentation of breast cancers. Most screening programs focus on early detection and rely on regular self or health worker breast examination with such services peaking in October of every year. Such screening is even less common in young women who are generally thought not to be at risk for breast cancer [6]. Again, more EOBCs presented with carcinoma-in-situ associated with invasive tumour (10.50% vs 4.30%). In line with the larger size at presentation, majority of EOBCs were thus at least pT2 (36.80%) vs 34.80% in the LOBC. In relation to lymph nodes, 13.80% of EOBCs were staged as pN3 compared to 9.0% of late onset cases. Further analysis also showed that majority of late onset cancers presented with no lymph node involvement (36.10%) compared with 34.50% in the EOBC. A larger frequency of positive lymph nodes (65.50% vs 63.00%) was recorded in EOBCs than in the older persons with a significant proportion being pN3 suggesting a higher stage at presentation of EOBCs compared to LOBCs although these findings are not statistically significant. In line with our findings, previous publications have reported larger tumours in younger patients with increased frequency of nodal involvement [6, 7, 11]. This could be attributed to the fact that, women under the age of 35 years do not routinely undergo breast cancer screening unless they are at high risk of developing breast cancer. Again, the sensitivity of a mammogram is equally low in this population due to the increased density of the breast, which obscures findings on mammogram [15]. The general late stage of presentation of our patients in both age groups has been established in the literature in relation to the presentation of breast cancer in Ghana [6].

The molecular subtypes used in this study are based on the recommended definition by the 13th St Gallen International Breast Cancer Conference (2013) Expert Panel review, defining Luminal A as (ER and PR positive, HER2 negative, Ki-67 low), Luminal B (HER2 negative) (ER-positive, HER negative and at least one of Ki-67 high or PR negative), Luminal B (HER2 positive) (ER-positive, HER2 positive and any of Ki-67 high or PR positive), HER2-enriched (HER2 positive, ER-negative, PR negative) and Triple Negative Breast Cancer (TNBC) (ER-negative, PR negative, HER2 negative). The panel also set the threshold of ≥ 20% as indicative of high Ki-67 [14]. Analysis of hormone receptor status indicates that in our setting, EOBCs more often tend to be hormone receptor (HR) negative when compared with LOBCs (47.70% vs 44.30%) contrary to the findings of Adrikopoulou et al. [4] in Greece who obtained 21.9% HR negative status among young women compared to 30.0% in the older women [4].

The Molecular subtypes of breast cancer in the current study are dominated by more TNBCs among EOBCs when compared to LOBCs (26.40% against 24.30%). TNBC is however the most frequent molecular subtype of breast cancer in both age groups. Luminal A breast cancer was also slightly more frequent in EOBC than in the older persons (11.30% vs 10.80%). The proportion of Luminal B with HER-2 Positive cancers was the same with both groups recording 9.40% while Luminal B with HER-2 Negative was more frequent in the older persons (LOBC) than in EOBC (22.10% vs 19.20%). HER-2-enriched cancers were also marginally more frequent in in older persons than in EOBCs in the study population (11.70% vs 11.10%). Elsewhere, a study by Collins et al. [16] that analysed the different subtypes of breast cancer in 399 women aged < 40 years identified 35% Luminal B tumours, 33% Luminal A tumours, 11% HER2 enriched tumours, and 21% TNBCs. Another study by Anders et al. [17, 18] evaluated gene expression profiles and identified 17% of tumours to be Luminal A, 27% Luminal B and 22% HER2 enriched cancers. In their study Basal-like tumours formed 34% of their cohort. Proportions in our study are closer to those found by Anders et al. with Luminal B (Luminal B HER2+ and Luminal B HER2−) forming 28.5% of tumours in our cohort. Our reported TNBC proportion of 26.40% though not including equivocal cases is significantly lower than the proportions reported in many earlier publications. With some literature quoting the percentage of TNBC to be up to 80% [19] but generally around 53.2% in the Ghanaian population it is likely that these rather high reported proportions are partly a result of false negatives due to poor pre-analytics [20]. Though our TNBC rates are lower than previously reported in the Ghanaian literature, it is still higher when compared to proportions of TNBC in other populations. Our finding is likely a result of improved pre-analytics because all cases were subjected to similar pre-analytical and analytical conditions.

## Conclusion

Our study reveals the high burden of breast cancer in women younger than 40 years in Ghana. It also confirms the reported adverse clinical and histopathological features of EOBC, though largely not statistically significant when compared with findings in LOBCs. The previously reported adverse outcome of EOBCs is likely a result of underlying molecular genetic abnormalities that require further study in our setting. Again, the nearly similar clinical and histopathological features of EOBCs and LOBCs in our setting confirms the generally reported aggressive nature of breast cancer in Africans. It also suggests that studying a cohort of EOBCs may provide molecular genetic insight into the general molecular biology of breast cancer in Africans. Such an effort will also improve the treatment and survival outcomes of not only young persons but all breast cancer patients.

### Limitations

This is a retrospective review that relied on data from the Electronic Medical Records System of reporting Pathology laboratories. Only available histopathology core data items could be retrieved for analysis. Detailed clinical and follow up data could not be assessed for the 2418 cases because this was not available on histopathology reports.

## Data Availability

All materials and data used in the study are available and can be provided as necessary by contacting the corresponding author.

## References

[1] GLOBOCAN 2020: Global Cancer Observatory. International Agency for Research on Cancer 2022. https://gco.iarc.fr/today/online-analysis-pie?v=2020&mode=cancer&mode_population=continents&population=900&populations=900&key=total&sex=0&cancer=39&type=0&statistic=5&prevalence=0&population_group=0&ages_group%5B%5D=0&ages_group%5B%5D=17&nb_items=7&group_cancer=1&include_nmsc=1&include_nmsc_other=1&half_pie=0&donut=0. Last accessed on 30th Nov 2022.

[2] Hironaka-Mitsuhashi A, Matsuzaki J, Takahashi R, Yoshida M, Nezu Y, Yamamoto Y, Shiino S, Kinoshita T, Ushijima T, Hiraoka N (2017). A tissue microRNA signature that predicts the prognosis of breast cancer in young women. PLoS ONE.

[3] Azim HA, Partridge AH (2014). Biology of breast cancer in young women. Breast Cancer Res.

[4] Andrikopoulou A, Chatzinikolaou S, Kyriopoulos I, Bletsa G, Kaparelou M, Liontos M, Dimopoulos M, Zagouri F (2022). The mutational landscape of early-onset breast cancer: a next-generation sequencing analysis. Front Oncol.

[5] Reyna C, Lee MC (2014). Breast cancer in young women: special considerations in multidisciplinary care. J Multidiscip Healthc.

[6] Naku Ghartey Jnr F, Anyanful A, Eliason S, Mohammed Adamu S, Debrah S (2016). Pattern of breast cancer distribution in Ghana: a survey to enhance early detection, diagnosis, and treatment. Int J Breast Cancer.

[7] Copson E, Eccles B, Maishman T, Gerty S, Stanton L, Cutress RI, Altman DG, Durcan L, Simmonds P, Lawrence G (2013). Prospective observational study of breast cancer treatment outcomes for UK women aged 18–40 years at diagnosis: the POSH study. J Natl Cancer Inst.

[8] Erić I, Petek Erić A, Kristek J, Koprivčić I, Babić M (2018). Breast cancer in young women: pathologic and immunohistochemical features. Acta Clin Croat.

[9] Siddig A, Tengku Din TADA-A, Mohd Nafi SN, Yahya MM, Sulong S, Wan Abdul Rahman WF (2021). The unique biology behind the early onset of breast cancer. Genes.

[10] Gómez-Flores-Ramos L, Álvarez-Gómez RM, Villarreal-Garza C, Wegman-Ostrosky T, Mohar A (2017). Breast cancer genetics in young women: what do we know?. Mutat Res Rev Mutat Res.

[11] Gómez-Flores-Ramos L, Castro-Sánchez A, Peña-Curiel O, Mohar-Betancourt A (2017). Molecular biology in young women with breast cancer: From tumor gene expression to DNA mutations. Rev Investig Clin.

[12] Kim J, Han W, Jung S-Y, Park YH, Moon H-G, Ahn SK, Lee JW, Kim MK, Kim JJ, Lee ES (2015). The value of Ki67 in very young women with hormone receptor-positive breast cancer: retrospective analysis of 9,321 Korean women. Ann Surg Oncol.

[13] Morrison D, Rahardja D, King E, Peng Y, Sarode V (2012). Tumour biomarker expression relative to age and molecular subtypes of invasive breast cancer. Br J Cancer.

[14] Goldhirsch A, Winer EP, Coates A, Gelber R, Piccart-Gebhart M, Thürlimann B, Senn H-J, Albain KS, André F, Bergh J (2013). Personalizing the treatment of women with early breast cancer: highlights of the St Gallen International Expert Consensus on the Primary Therapy of Early Breast Cancer 2013. Ann Oncol.

[15] Gabriel CA, Domchek SM (2010). Breast cancer in young women. Breast Cancer Res.

[16] Collins L, Marotti J, Gelber S, Cole K, Ruddy K, Kereakoglow S, Brachtel E, Schapira L, Come S, Winer E (2012). Pathologic features and molecular phenotype by patient age in a large cohort of young women with breast cancer. Breast Cancer Res Treat.

[17] Anders CK, Acharya CR, Hsu DS, Broadwater G, Garman K, Foekens JA, Zhang Y, Wang Y, Marcom K, Marks JR (2008). Age-specific differences in oncogenic pathway deregulation seen in human breast tumors. PLoS ONE.

[18] Anders CK, Hsu DS, Broadwater G, Acharya CR, Foekens JA, Zhang Y, Wang Y, Marcom PK, Marks JR, Febbo PG (2008). Young age at diagnosis correlates with worse prognosis and defines a subset of breast cancers with shared patterns of gene expression. J Clin Oncol.

[19] Jiagge EM, Ulintz PJ, Wong S, McDermott SP, Fossi SI, Suhan TK, Hoenerhoff MJ, Bensenhaver JM, Salem B, Dziubinski M (2021). Multiethnic PDX models predict a possible immune signature associated with TNBC of African ancestry. Breast Cancer Res Treat.

[20] Lukong KE, Ogunbolude Y, Kamdem JP (2017). Breast cancer in Africa: prevalence, treatment options, herbal medicines, and socioeconomic determinants. Breast Cancer Res Treat.

